# Case of Tubercular Phthisis, with Remarks

**Published:** 1848-06

**Authors:** John R. Buck

**Affiliations:** Louisville, Ky.


April, 1848.

---

### Art. III.—*Cases of Tubercular Phthisis, with Remarks.* By John R. Buck, M.D., of Louisville, Ky.

Tubercular disease of the lungs is so generally regarded as incurable, that many physicians are strongly disinclined to undertake its treatment; indeed, it is often declared to be prejudiced by any treatment, and patients are advised to avoid both physicians and medicines. The failure to recognise this disease in the early stages, previous to the softening of tubercular matter, is doubtless the cause to which is mainly referable the opinion that it can neither be cured nor stayed in its progress. If it is recognised before the foreign matter (for so we must regard tubercle) excites that degree of inflammation in the surrounding structures as to render suppuration inevitable, I entertain no doubt but that the disease may generally be arrested and frequently cured. The object of this communication, however, is not to discuss the curableness of phthisis in its different stages, but merely to give a very brief and condensed report of several cases, to show that it may be benefitted and sometimes entirely arrested, not to say cured, after tubercle has softened and been expectorated.

Mrs. F., æt. 31 years, requested my attendance in December 1842. Her father, two brothers, and a sister, had died of tubercular consumption; also her husband; since that time another sister has died of the same disease. She is tall, very spare, with dark auburn hair and beautiful, clear, white skin, She had not been confined to her bed at all, but had been much debilitated for some months, and within the last few weeks previous to my seeing her, had had more or less dry short cough, shooting pains through the chest, with very slight fever in the evening. I found slight dullness at the summit of the left lung, with increased resonance of the voice and feebleness of the continuous murmur; no other evidence of disease in any part of the chest. In a few weeks softening of tubercular matter occurred as shown by the general symptoms and physical signs, as well as by the presence of tubercle in the expectoration. Local depletion was practiced several times at the summit of the chest anteriorly and posteriororly; after which an issue was established in the same vicinity and kept open for six months,—discharging freely the greater portion of the time: so soon as the issue began to discharge freely, she was placed upon a syrup of the iodide of iron (ʒi. of the iodide to ℥i. of pure syrup); of this a tea spoonful was given morning, noon, and night, one hour before each meal. This was the general plan of treatment. In May 1843, she had so improved that I ceased to attend her: since that time (it is now April, 1848) she has had no return of the pulmonary disease whatever, and is in good health.

H. D., æt. 46. I first visited this patient in January 1845. There was no family predisposition to phthisis. In 1831 he had profuse hemoptysis coming on suddenly, while talking in an ordinary tone of voice to some friends, and at a time when he thought himself in perfect health; this returned occasionally for a month or six weeks, since when, there has never been the slightest appearance of blood in the expectoration; his general health continuing good until December 1844, when he contracted catarrh. When I first saw him, he was somewhat emaciated, complained of great weakness, loss of appetite, cough, night sweats, hectic fever, and frequent chilliness. At the summit of the right lung, and for two inches below the clavicle, there was well marked dullness. Bronchial respiration and broncophony in the same limits.

The application of cups below the right clavicle seemed for a time to arrest the disease entirely; in a few weeks however, having been slightly exposed in bad weather, he was taken with increased cough, shooting pains through the chest, and more febrile action; this train of symptoms was followed in a few weeks by distinct chills, copious night sweats, cavernous rhonchus, and tuberculous expectoration. An issue was established one inch below the clavicle with the Vienna paste, and the following prescription made:

℞. Quiniæ Sulphatis, ℈ij.
Hyoscyami Ext. grs. x.
M. ft. Pil. No. 20.

Two pills every night at bed time.

℞. Ferri. Iodid. Syrupi, ℥vj.

A teaspoonful one hour before each meal, in a little flaxseed or slippery-elm mucilage.

To relieve nausea and eructations which were occasionally troublesome during the course of the disease, the following was given.

℞. Argenti Oxyd. ℈j.
Acaciæ Mucilaginis, q. s.
Ft. Pil. No. 20.

One pill morning and night.

The whole body was thoroughly sponged with alcohol at bed time every night. This patient was discharged about the middle of April, having regained his strength, and flesh, and being to all appearance free from disease.

W. I., æt. 22. consulted me first in the summer of 1844. He had been examined by several eminent physicians of New Orleans, all concurring in the opinion that his lungs were tuberculous; he had had repeated attacks of hemorrhage from the lungs,—had several after he came under my treatment. All the physical signs as well as general symptoms of tubercular disease of the lungs were well marked. The same general plan of treatment was adopted in this case, and with the same result:—the young man recovered and is now to all appearance, and as he feels, in perfect health.

It must not be supposed however, that I have been thus fortunate in all such cases, or even in a majority that have come under my care; not so; a very small proportion only have terminated favorably, and I now enter upon the treatment of phthisis, after the tubercle begins to soften with but little hope of very materially arresting its progress, and none of curing it. If, however, it is even possible to arrest the disease in this advanced stage, it is not only important to know it, that we may give every patient the benefit of this possibility, but it also teaches us that in the earlier stages we may with some confidence hope for a permanent cure.

April, 1846.

